# Multicenter Randomized Trial of Methylprednisolone vs. Intravenous Immunoglobulins to Treat the Pediatric Inflammatory Multisystem Syndrome—Temporally Associated With SARS-CoV-2 (PIMS-TS): Protocol of the Swissped RECOVERY Trial

**DOI:** 10.3389/fped.2022.905046

**Published:** 2022-05-20

**Authors:** Tatjana Welzel, Nina Schöbi, Maya C. André, Douggl G. N. Bailey, Geraldine Blanchard-Rohner, Michael Buettcher, Serge Grazioli, Henrik Koehler, Marie-Helene Perez, Johannes Trück, Federica Vanoni, Petra Zimmermann, Andrew Atkinson, Carlos Sanchez, Elizabeth Whittaker, Saul N. Faust, Julia A. Bielicki, Luregn J. Schlapbach, the Swissped Recovery Trial

**Affiliations:** ^1^Paediatric Research Center, University Children's Hospital Basel, University of Basel, Basel, Switzerland; ^2^Paediatric Pharmacology and Pharmacometrics, University Children's Hospital Basel, University of Basel, Basel, Switzerland; ^3^Division of Paediatric Rheumatology, University Children‘s Hospital Basel, University of Basel, Basel, Switzerland; ^4^Division of Paediatric Infectious Diseases, Department of Paediatrics, Inselspital, Bern University Hospital, University of Bern, Bern, Switzerland; ^5^Division of Respiratory and Critical Care Medicine, University Children‘s Hospital Basel, University of Basel, Basel, Switzerland; ^6^Department of Paediatric Hematology and Oncology, University Children's Hospital, Eberhard Karls University, Tuebingen, Germany; ^7^Paediatric and Neonatal Intensive Care Unit, Children's Hospital of Eastern Switzerland, St. Gallen, Switzerland; ^8^Paediatric Immunology and Vaccinology Unit, Division of General Paediatrics, Department of Child, Woman and Adolescent Medicine, Faculty of Medicine, Geneva University Hospitals, Geneva, Switzerland; ^9^Paediatric Infectious Diseases Unit, Department of Paediatrics, Cantonal Hospital Lucerne, Lucerne, Switzerland; ^10^Division of Neonatal and Paediatric Intensive Care, Department of Child, Woman and, Adolescent Medicine, Faculty of Medicine, Geneva University Hospitals, Geneva, Switzerland; ^11^Department of Paediatrics, Cantonal Hospital Aarau, Aarau, Switzerland; ^12^Paediatric Intensive Care Unit, University Hospital Lausanne, Lausanne, Switzerland; ^13^Division of Immunology and Children‘s Research Center, University Children's Hospital Zurich, University of Zurich (UZH), Zurich, Switzerland; ^14^Clinic of Paediatrics, Paediatric Institute of Southern Switzerland, Ente Ospedaliero Cantonale (EOC), Bellinzona, Switzerland; ^15^Faculty of Biomedical Sciences, Università della Svizzera italiana, Lugano, Switzerland; ^16^Department of Community Health, Faculty of Science and Medicine, University of Fribourg, Fribourg, Switzerland; ^17^Department of Paediatrics, Fribourg Hospital, Fribourg, Switzerland; ^18^Infectious Diseases Research Group, Murdoch Children's Research Institute, Parkville, VIC, Australia; ^19^Section of Paediatric Infectious Diseases, Imperial College London, London, United Kingdom; ^20^Department of Paediatric Infectious Diseases, Imperial College Healthcare NHS Trust, London, United Kingdom; ^21^NIHR Southampton Clinical Research Facility and Biomedical Research Centre, University Hospital Southampton NHS Foundation Trust, Southampton, United Kingdom; ^22^Faculty of Medicine and Institute for Life Sciences, University of Southampton, Southampton, United Kingdom; ^23^Centre for Neonatal and Paediatric Infection, St George's University, London, United Kingdom; ^24^Department of Intensive Care and Neonatology, Children‘s Research Center, University Children's Hospital Zurich, Zurich, Switzerland; ^25^Paediatric Intensive Care Unit, Queensland Children‘s Hospital and Child Health Research Centre, The University of Queensland, Brisbane, QLD, Australia

**Keywords:** children, COVID-19, SARS-CoV-2, mortality, MIS-C, quality of life, treatment, trial

## Abstract

**Introduction:**

In 2020, a new disease entitled Pediatric Inflammatory Multisystem Syndrome temporally associated with COVID-19 (PIMS-TS), or Multisystem Inflammatory Syndrome in Children (MIS-C), emerged, with thousands of children affected globally. There is no available evidence based on randomized controlled trials (RCT) to date on the two most commonly used immunomodulatory treatments, intravenous immunoglobulins (IVIG) and corticosteroids. Therefore, the Swissped RECOVERY trial was conducted to assess whether intravenous (IV) methylprednisolone shortens hospital length of stay compared with IVIG.

**Methods and Analysis:**

Swissped RECOVERY is an ongoing investigator-initiated, open-label, multicenter two-arm RCT in children and adolescents <18 years hospitalized with a diagnosis of PIMS-TS. The trial is recruiting at 10 sites across Switzerland. Patients diagnosed with PIMS-TS are randomized 1:1 to methylprednisolone IV (10 mg/kg/day for 3 days) or IVIG (2 g/kg as a single dose). The primary outcome is hospital length of stay censored at day 28, death, or discharge (whichever is first). The target total sample size is ~80 patients 1:1 randomized to each study arm. Ancillary and exploratory studies on inflammation, vaccination acceptance and coverage, long-term outcomes, and healthcare costs are pre-planned.

**Significance:**

Currently, robust trial evidence for the treatment of PIMS-TS is lacking, with a controversy surrounding the use of corticosteroids vs. IVIG. This trial will provide evidence for the effectiveness and safety of these two treatments.

**Ethics and Dissemination:**

The study protocol, which was designed based on the U.K. RECOVERY trial, the patient information and consent forms, and other study-specific study documents were approved by the local ethics committees (Project ID: 2021-00362).

**Registration Details:**

The study is registered on the Swiss National Clinical Trials Portal (SNCTP000004720) and Clinicaltrials.gov (NCT 04826588).

## Introduction

In April 2020, within weeks of the global spread of the severe acute respiratory syndrome coronavirus 2 (SARS-CoV-2) pandemic, reports of pediatric patients with a new hyperinflammatory multisystemic condition emerged from different countries (1, 2). Patients manifested clinical and laboratory signs of inflammation with fever, high C-reactive protein and ferritin serum levels, coupled with altered function of one or several organ systems, in presence of confirmed or suspected previous exposure to or infection with SARS-CoV-2 (3). Different, albeit largely overlapping, case definitions have been used globally, including Pediatric Inflammatory Multisystem Syndrome temporally associated with COVID-19 (PIMS-TS) and Multisystem Inflammatory Syndrome in children associated with COVID-19 (MIS-C) (4–6). Patients with PIMS-TS may require Pediatric Intensive Care Unit (PICU) admission and organ support, with a mortality rate of around 2% in high-income countries and up to 9% in middle-income countries (7, 8). Since the first case reports, epidemiological and biochemical characterization of PIMS-TS has made enormous progress resulting in an improved understanding of the clinical phenotypes and underlying immunological mechanisms (9–13).

According to several expert opinion-based consensus guidelines, anti-inflammatory treatment represents the mainstay of management (14–17). Intravenous corticosteroids, such as methylprednisolone, and intravenous immunoglobulins (IVIG) are the most widely recommended therapeutic interventions (18). However, observational data from large cohorts, some propensity-matched, provide conflicting results (19–21). To date, no randomized controlled trials (RCTs) have been published, resulting in a lack of clinically directive data about intravenous (IV) methylprednisolone vs. IVIG in children with PIMS-TS. Given expected challenges for accessing IVIG in many global regions with very young populations and therefore large numbers of children at risk, evidence to support or refute the importance of IVIG for PIMS-TS treatment is urgently needed.

We therefore designed a randomized controlled multicenter Swissped RECOVERY trial to assess the effectiveness of IV methylprednisolone vs. IVIG in hospitalized PIMS-TS patients. The trial design was informed by the adaptive trial on children with PIMS-TS in the UK (https://www.recoverytrial.net/files/recovery-pediatric-sap-v1-1.pdf) (22), which is ongoing as part of the Randomized Evaluation of COVID-19 Therapy (RECOVERY) project (https://www.recoverytrial.net/). Here we describe the Swissped RECOVERY trial protocol.

## Methods and Analysis

### Study Design

**Figure 1:**
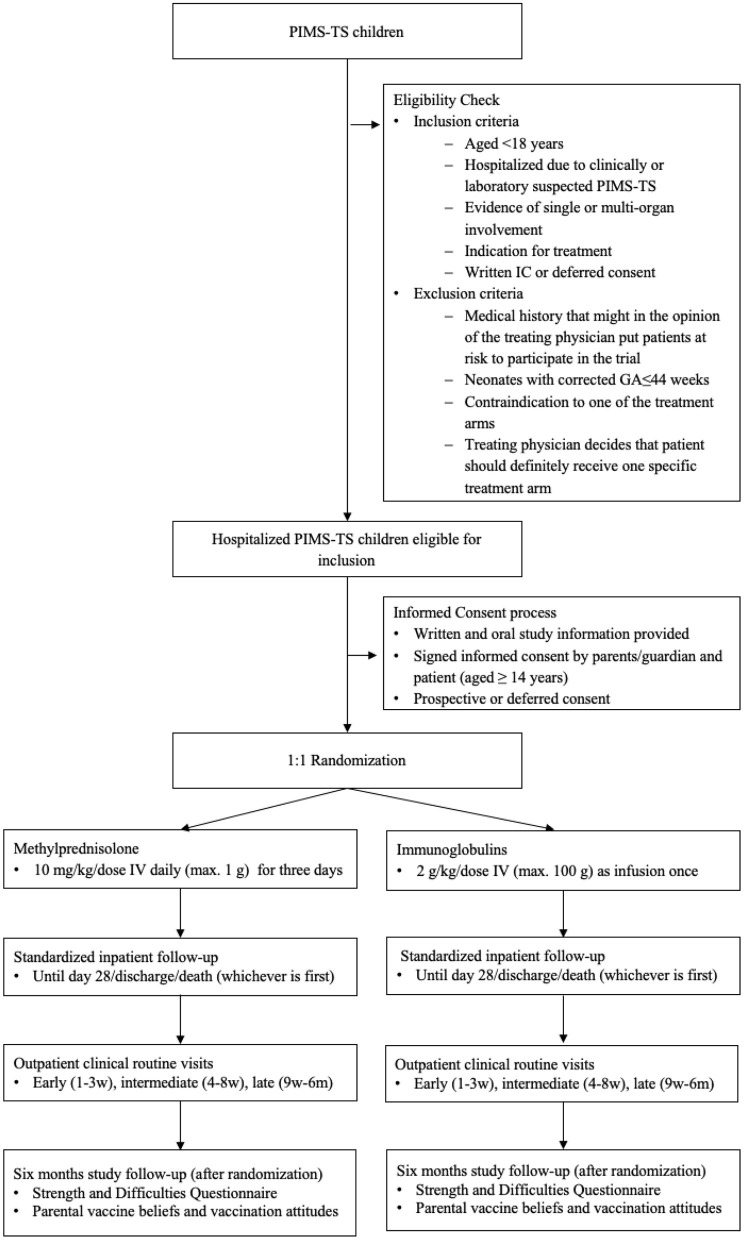
Study flow chart. PIMS-TS, Pediatric Inflammatory Multisystem Syndrome temporally associated with COVID-19; IC, informed consent; GA, gestational age; mg, milligram; kg, kilogram; IV, intravenous; g, gram; w, week; m, month.

Swissped RECOVERY is an investigator-initiated randomized multicenter open-label two-arm trial in children and adolescents hospitalized with PIMS-TS. Children and adolescents diagnosed with PIMS-TS in accordance to the Best Practice Recommendations for the Diagnosis and Management of PIMS-TS in Switzerland (17), are randomized 1:1 to IV methylprednisolone (10 mg/kg/day for 3 days) or IVIG (2 g/kg as a single dose). Follow-up data is collected from all study participants until 6 months after randomization. The trial flow chart is presented in Figure 1.

Swissped RECOVERY was inspired by RECOVERY trial running in the United Kingdom (NCT04381936; U.K. RECOVERY). The U.K. RECOVERY Trial was conceived by lead investigators Professors Martin Landray and Peter Horby, University of Oxford. The protocol (including inclusion/exclusion criteria and outcomes) of the Swissped RECOVERY trial is aligned with the RECOVERY trial to enable future joint analyses. The case report forms (CRFs) of Swissped RECOVERY include but are not limited to data captured in the U.K. RECOVERY study, allowing future data pooling. A key difference is the omission of the “standard of care (SOC)” arm (meaning no additional anti-inflammatory treatment) in the Swissped RECOVERY trial. This was agreed by all stakeholders based on: i) an increasing number of observational studies demonstrating effectiveness of anti-inflammatory treatment, and ii) the consideration that there was no equipoise for the “IVIG” or “methylprednisolone” arm compared to the “SOC” arm (19–21). Therefore, different to the U.K. RECOVERY trial, Swissped RECOVERY was simplified before the randomization of the first patient to a two-arm trial, with study allocation limited to a 1:1 randomization to IV methylprednisolone (10 mg/kg/dose for 3 days) or IVIG (2 g/kg as single dose) throughout the trial conduct.

### Trial Setting

Swissped RECOVERY recruits in pediatric emergency departments (EDs), pediatric wards, and PICUs of 10 participating sites across Switzerland. The participating sites are secondary and tertiary pediatric hospitals with experience in PIMS-TS management, supported by local SwissPedNet (www.swisspednet.ch) infrastructure, and are located in Aarau, Basel, Bern, Bellinzona, Fribourg, Geneva, Lausanne, Lucerne, St. Gallen, and Zurich. Site principal investigators (PIs) are qualified by education and experience to assume responsibility for the proper conduct of the trial at each study site.

### Participants

Hospitalized patients aged <18 years with clinical and/or laboratory evidence for PIMS-TS in line with the Swiss PIMS-TS guidelines which apply to the RCPCH case definition (4, 17), and considered to require anti-inflammatory treatment, are eligible. Patients, who, in the opinion of the attending clinician, have a medical history that may put the patient at significant risk in case of study participation, or in case of a specific contra-indication to one of the active treatments, or where the treating physician decides that a specific active treatment has to be administered, are excluded. Neonates with a corrected gestational age of ≤44 weeks are also excluded. Details of inclusion and exclusion criteria are provided in Table 1.

**Table 1:** Inclusion and exclusion criteria.

**Rule**	**Criterion**
Inclusion	• Aged <18 years
	• Hospitalized due to clinically or laboratory suspected PIMS-TS
	• Evidence of single or multi-organ involvement • Clinician decides to treat for PIMS-TS • Written informed consent
Exclusion	• Medical history that might, in the opinion of the clinician, put the patient at risk to participate in the trial • Neonates with a corrected gestational age of ≤ 44 weeks • Specific contraindication to one of the treatment arms • Decision of the attending clinician that the patient should definitely receive one of the treatment arms

*PIMS-TS, Pediatric Inflammatory Multisystem Syndrome temporally associated with COVID-19*.

### Screening, Recruitment, and Informed Consent (IC) Process

Potential study participants are screened for eligibility in the participating EDs, wards, and PICUs by the respective clinical and research teams. Eligibility is reviewed and documented by a trained and qualified member of the study team. The screening-enrolment process is documented in the screening-enrolment log. Written and oral study information (available in Italian, French, English, and German) is provided to the parents/guardians of the potential study participant and the patient themselves if aged ≥11 years. The parent/guardian and patient are informed that the trial participation is voluntary and that they can refuse or withdraw their consent to participate at any time and for any reason without negative consequences. In addition, the parent/guardian and the patient are informed about, and formally asked to, consent to re-use of health-related data from this trial for other research projects.

The written IC is obtained from parents/guardians of the patient (children/adolescents <18 years), and the patient themselves, if aged ≥14 years. If timely IC is not feasible (if parents or a suitable relative are not present immediately to provide consent despite reasonable efforts to obtain consent by the study team), the study team can use a deferred consent approach, e.g., an independent doctor can declare the patient's suitability for trial participation (23). In that case, written IC by the parents/guardians and patients is sought as soon as possible. If the IC is withdrawn, or is not given after deferred consent, the data are used until the time of withdrawal to assure the scientific quality of the trial.

### Randomization and Blinding

After successful enrolment, a dedicated person from each study site enters the encoded patient‘s enrolment data into the online electronic data capture (EDC) system REDCap^TM^. Randomization occurs online in a 1:1 ratio to IV methylprednisolone vs. IVIG. The randomization list is computer-generated based on random permuted blocks size of 30 by the trial data manager, meaning the assignment is repeated from the 31st patient. Having confirmed eligibility and availability of IC, the allocated treatment arm is displayed on the screen. Due to the challenges in blinding two drugs with different volumes and frequency of administration, and due to the pragmatic study design, the study is open-labeled.

### Trial Treatment Arms

After randomization, the clinician in charge prescribes the allocated treatment and ensures timely administration. Study treatment is given through an existing peripheral or central intravenous catheter as follows:

- *Steroids:* Methylprednisolone 10 mg/kg/dose (maximum dose 1,000 mg per day) for 3 days once daily. This is similar to the approach in the Swiss PIMS-TS guidelines (17), international consensus statements, and in line with the U.K. RECOVERY trial.
- *Immunoglobulins:* IVIG 2 g/kg/dose (maximum dose 100 g) as a single dose given as a slow infusion in line with the hospital standard operating procedures (infusion duration 12 ± 4 h). This is based on the Swiss PIMS-TS guidelines (17), the 2021 American College of Rheumatology guidelines (14), Kawasaki disease guidelines including the European SHARE initiative (24), the American Heart Association guidelines (25) and in line with the U.K. RECOVERY trial.

Established SOC treatments (e.g., fluid management, respiratory support, inotropes, and/or anticoagulation including acetylsalicylic acid) and use of antimicrobials is uncontrolled and open to the attending clinician or hospital guidelines. Additional immunomodulative treatment, can be administered at the discretion of the responsible clinical team, and are captured in the study database. Additional immunomodulative treatment include administration of i) the non-randomized treatment (IVIG/methylprednisolone), ii) a second dose/increased dose of the randomized treatment (IVIG/methylprednisolone), iii) the randomized treatment (methylprednisolone) for a longer period and iv) biological treatment such as Interleukin (IL)-1 inhibition, IL-6 inhibition, or tumor necrosis factor (TNF) inhibitors. Sites are encouraged to observe patients for a minimum of 24 h before considering additional immunomodulatory/biological treatments, unless there is a clear clinical indication for escalation.

### Data Collection Including Study Follow-Up

**Figure 2:**
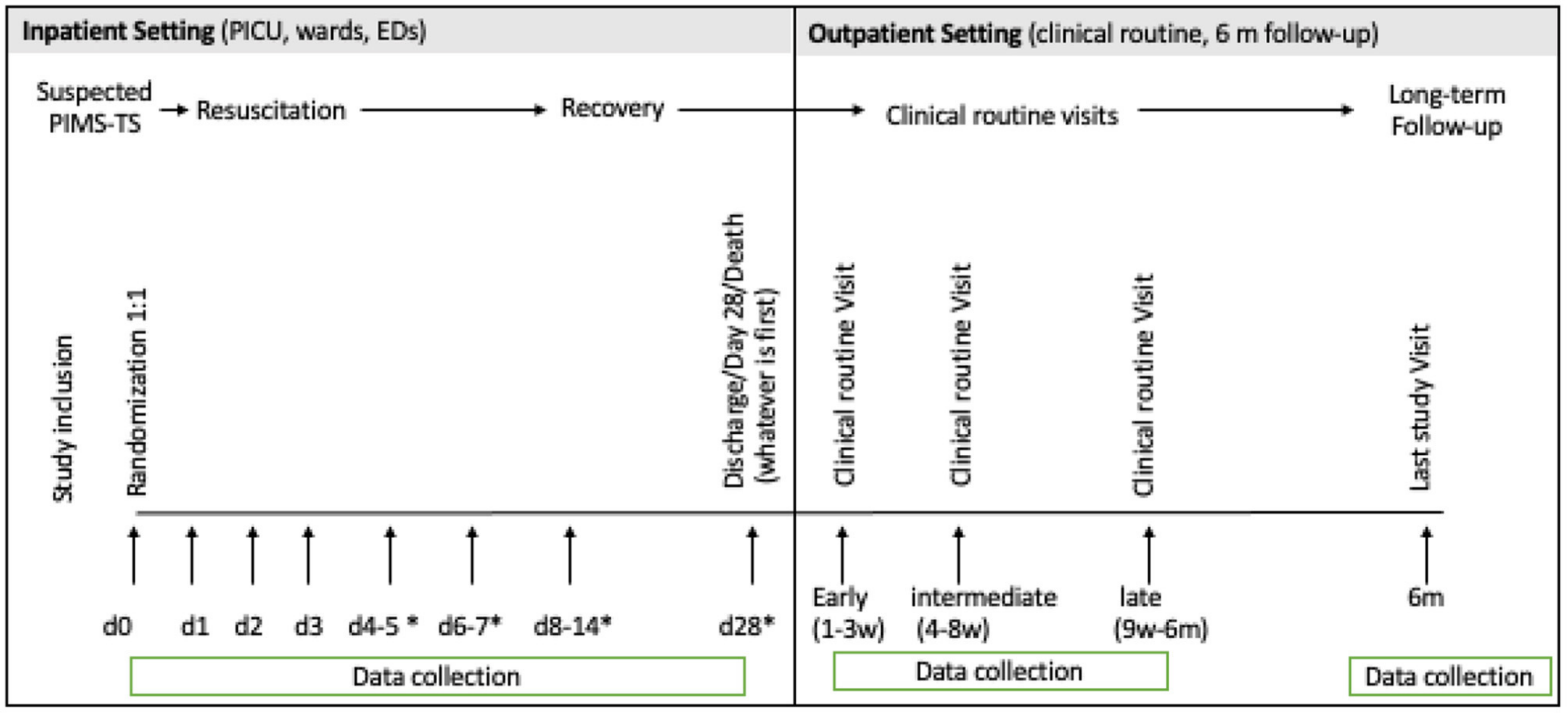
Study prodedures. PICU, Pediatric Intensive Care Unit; ED, Emergency Department; d, day; w, week; m, month. ^*^if still hospitalized.

Data on length of hospital stay, as well as persistent PIMS-TS symptoms, vital signs, organ support, laboratory parameters, biomarkers, and study treatments including the randomized trial treatment as well as additional immunomodulatory treatments are collected prospectively in all patients daily for day 0, 1, 2, 3, and then for day 4–5, 6–7, and 8–14 or until discharge. Follow-up data is assessed until 6 months after randomization at defined time points (Figure 2; Tables 2, 3).

**Table 2:** Standarized data collection and follow-up inpatients.

**Days**	**0**	**1**	**2**	**3**	**4–5**	**6–7**	**8–14**	**Death/discharge/28 days**
					**If hospitalized**			
**Anamnestic data**								
Demographics	x							
Patient‘s medical history	x							
Sars-CoV-2 exposure	x							
PIMS-TS symptoms	x							
**Clinical assessment**								
Vital signs		x	x	x	x	x	x	x
Central capillary refill time		x	x	x	x	x	x	x
Level of consciousness		x	x	x	x	x	x	x
**Data collection of disease course and need for additional support/PICU**								
PICU admission								x
Respiratory support								x
Cardiac support								x
Bleeding								x
Last day of fever (≥ 38°C)								x
First day with CRP <50 mg/l								x
Final diagnosis								x
**Cardiologic assessment**								
Echocardiogram	x	x	x	x	x	x	x	x
Echocardiography	x	x	x	x	x	x	x	x
**Laboratory assessment**								
Hematology	x	x	x	x	x	x	x	x
Inflammatory parameters	x	x	x	x	x	x	x	x
Biochemistry	x	x	x	x	x	x	x	x
Microbiology testing	x	(x)	(x)	(x)	(x)	(x)	(x)	(x)
**Treatment**								
Trial treatment								x
Supportive treatment								x
Additional (rescue) treatment								x

**Table 3:** Data assessment according study schedule.

**Trial phase**	**Enrolment**	Inpatient follow-up^*^	**Last inpatient visit^a^**	**Outpatient follow-up**	
				**Clinical routine**	**6 months**
**Trial participation**					
Eligibility screening	X				
Written and oral study information	X°				
Signed informed consent (IC)	X°				
Randomization	After IC				
Administration of trial treatment	After randomization				
**Assessments**					
Demographics		X			
Medical history		X		X	
Clinical assessment		X	X	X	
Cardiology assessment		(X)	(X)	(X)	
Laboratory assessment		(X)	(X)	(X)	
Supportive treatment			X	X	
Additional (rescue) treatment			X	X	
Specified severe adverse events		X	X		
Concomitant care/Healthcare utilization			X	X	
SDQ questionnaire					X
Vaccine questionnaire					X

*X**°** Witnessed or deferred consent may be used in exceptional cases*.

* *See Table 2 for details*.

a *Discharge, death, day 28—whatever is first*.

*(X) performed examinations based on clinical decisions, data collection if available*.

After discharge, study follow-up is in line with clinical routine-monitoring scheduled as recommended for PIMS-TS patients in Switzerland (17). Clinical routine follow-up includes a review of the clinical course, physical and laboratory examinations, and specific examinations such as electrocardiogram (ECG), echocardiogram, and additional immunological tests if indicated. In addition, behavioral/functional status is assessed in the 6th month after randomization by the Strengths and Difficulties Questionnaire (SDQ) (26). In addition, a vaccination questionnaire is provided to the parents/guardians of the study participant. If no clinical routine follow-up is indicated at the 6 months follow-up, the patients and/or their parents/guardians are contacted by the study team by phone or mail for completion of the questionnaires. Relevant study data for each enrolled study participant are recorded *via* REDCap^TM^.

### Outcome Measures

The primary study outcome is to assess the differences between hospital length of stay for the two trial arms, defined as the time in days between admission and discharge (or death, if sooner, or 28 days, if the patient is still hospitalized). In supplementary analyses, time from randomization to discharge will also be considered. Secondary outcomes include all-cause mortality, indication and duration of organ support, and proportion of cardiac pathologies. Safety outcomes include safety events and proportion, indication and duration of rescue treatment. In addition, exploratory analyses including time-to event analyses are planned (Table 4). Long-term outcomes include follow-up data with focus on ongoing PIMS-TS symptoms and fatigue as well as behavioral/functional status (Table 4). Ancillary study outcomes include assessment of parental vaccination beliefs and vaccination coverage in the PIMS-TS patient themselves and families of PIMS-TS patients. Long-term outcomes, and ancillary study outcomes will be investigated and reported separately from the main analyses.

**Table 4:** Outcomes assessed.

**Outcome**	**Criterium**	**Definition**
Primary outcome*	Hospital length of stay	• Duration of hospital stay (days), censored at 28 days/death/discharge (whichever is first). *Note:* • If a patient is discharged from hospital and later readmitted, this is documented as an SAE. • If a patient is transferred from one hospital to another hospital, the total duration of hospitalization is calculated.
Secondary outcomes*	Effectiveness	• All-cause mortality after randomization, censored at 28 days/death/discharge • Proportion of patients requiring (i) inotrope support, (ii) respiratory support, (iii) Extracorporeal Membrane Oxygenation (ECMO), and/or (iv) renal replacement censored at 28 days/death/discharge • Overall • Post-baseline: new after randomization • Duration of (i) inotrope support, (ii) respiratory support, (iii) Extracorporeal Membrane Oxygenation (ECMO), and/or (iv) renal replacement censored at 28 days/death/ discharge • Overall • Post-baseline: new after randomization • Proportion of patients with cardiac pathologies based on (i) echocardiography and/or (ii) electrocardiogram censored at 28 days/death/ discharge • Overall • Post-baseline: new after randomization
Safety outcomes	Safety	• Descriptive reporting of severe adverse events (SAE) observed in the trial • Proportion of patients with (i) major bleeding, (ii) thrombotic events censored at 28 days/death/discharge • Overall • Post-baseline: new after randomization
Exploratory outcomes	Proxy measures of intervention efficacy	• Proportion of patients receiving rescue treatment censored at 28 days/death/discharge • Overall • Post-baseline: new after randomization • Duration of rescue treatment censored at 28 days/death/ discharge • Proportion of patients receiving rescue treatment censored at 28 days/death • Overall • Post-baseline: new after randomization • Duration of rescue treatment censored at 28 days/death/ discharge • Proportion of patients receiving indicated rescue treatment by a blinded review committee censored at 28 days/death/discharge • Proportion of patients with admission (i) to Pediatric Intensive Care Unit (PICU) or/and (ii) ward censored at 28 days/death/ discharge • Overall • Post-baseline: new after randomization • Duration of length of stay at (i) PICU and /or (ii) ward censored at 28 days/death/discharge • Overall • Post-baseline: new after randomization • Time to normalization of inflammation
Long-term outcome	Follow-up	• Portion of patients (N, %) with (i) ongoing PIMS-TS symptoms, fatigue, or other musculoskeletal symptoms • Behavioral/functional status at 6 months after randomization, assessed by the Strengths and Difficulties Questionnaire • Proportion of patients with cardiac pathologies based on (i) echocardiography and/or (ii) electrocardiogram at early, intermediate and late follow-up
Anxillary study	Vaccination	• Proportion of (i) patients and (ii) parents vaccinated against SARS CoV-2 before PIMS-TS diagnosis and 6 months thereafter • Proportion of patients admitted to PICU or ward when being diagnosed as PIMS-TS with respect to vaccine status: (i) complete, (ii) one vaccination, (iii) no vaccination • Duration of hospital stay (days) on PICU or ward when being diagnosed as PIMS-TS with respect to vaccine status: (i) complete, (ii) one vaccination, (iii) no vaccination • Attitudes and beliefs of parents toward vaccination against SARS CoV-2 before and after PIMS-TS diagnosis of their child • Vaccination reactions compared to general age-merged population from other studies

The primary and secondary outcomes, marked with an asterisk (*), are based on the U.K. RECOVERY trial and its statistical analysis plan. All other outcomes are exploratory and performed in the Swissped RECOVERY trial only.

### Quality Control and Electronic Data Capture

The study coordination provides study training for site staff. Written standard operating procedures and a manual of procedures are provided to each study site. Site initiation visits (SIV) were performed to deliver trial-specific training of key clinical personnel and the site study team. In addition, regular videoconferencing takes place to ensure good quality study conduct.

Study data are captured in REDCap^TM^, a web-based database (27), hosted by the University Children's Hospital Basel. Data are stored on a firewall-protected server and regularly backed up. Electronic access is strictly controlled using role-based access permissions defined by the local REDCap^TM^ administrator and by the PIs. All data modifications can be identified through a complete audit trail.

### Monitoring

Training and delegation logs have been monitored for completeness. Good Clinical Practice (GCP) certificates of the PIs have been checked to be up-to-date before trial start. In addition, during the trial, regular monitoring is conducted in line with a pre-agreed monitoring plan. Randomization and data entry, data consistency, missing data points, or possible errors in the database REDCap^TM^ are reviewed centrally by the trial data manager. In accordance with the International Council for Harmonization of Technical Requirements for Pharmaceuticals for Human Use (ICH) and GCP, guideline audits may be performed by competent ethics committees (CEC) and other competent authorities during the study course. PIs allow trial-related monitoring, including audits, CEC review, and regulatory inspections by providing direct access to source data and all documents required.

### Trial Governance and DSMB

A Trial Steering Group (TSG) was formed comprising the sponsor-investigator, other lead investigators, the trial manager, and members of the Pediatric Research Centre at the University Children's Hospital Basel. The TSG is responsible for the day-to-day running, trial management, and planning of regular meetings with all PIs. To effectively interact with the U.K. RECOVERY study, the Swiss TSG has regular meetings with experts from the RECOVERY trial group.

An Independent Data Monitoring Committee (IDMC) composed of three members, namely i) Dr. Michelle Clements, MRC Clinical Trials Unit at UCL, United Kingdom ii) Dr. Carlo Giaquinto, Full Professor of Pediatrics, University of Padova, Italy, iii) Dr. Robin Kobbe, University Medical Centre Hamburg-Eppendorf, Germany. The IDMC is responsible to safeguard the interests of trial participants, to assess the safety, and to contribute to the monitoring of the overall conduct of the Swissped RECOVERY trial. It is the only instance allowed to see confidential accumulating data for the trial by randomized arm. The IDMC monitors evidence for treatment harm [e.g., no equipoise of trial treatment arms, severe adverse events (SAE)] and will make recommendations on continuation of the trial or discontinuation for safety reasons. Details of IDMC functioning and procedures are specified in the IDMC Charter agreed and signed by all IDMC members.

### Safety Reporting

We will report on SAE which are likely to be related to the trial treatments. Anticipated events, including SAEs, that are either efficacy endpoints, consequences of the underlying disease, and common events which are the consequence of conditions preceding randomization will be exempted from expedited reporting. If an AE occurs, the PI assesses whether this is an SAE against the protocol. An SAE form is completed and the Sponsor-Investigator is notified within 24 h of the PI becoming aware, if the SAE requires expedited reporting. The Sponsor-Investigator re-evaluates the SAE and follows the CEC notification requirements. The participants are followed-up until resolution or stabilization of the SAE.

In addition, the CEC and regulatory authorities receive annual safety reports and are informed about study stop/end in agreement with local requirements. The Sponsor-Investigator may terminate the study prematurely according to certain circumstances (e.g., early evidence of benefit or harm of the treatment arms, ethical concerns).

### Sample Size Calculation and Power

The larger the number of participants randomized, the more accurate the results will be, but the numbers that can be randomized will depend critically on the progress of the epidemic and the influence of vaccination on children/family groups. For the period of the trial, it is estimated that between ~50 and 120 children can be recruited across the 10 centers. According to expert opinion, the best estimate (i.e., the mode) would be ~80 children in total, assigned with 1:1 randomization. Accordingly, for the primary outcome comparing mean log-transformed length of stay for those on methylprednisolone (*N* = 40) to those on IVIG (*N* = 40) using a two-sided *t*-test and with a 5% significance level, we would be able to detect a normalized effect size of ~0.65 with 80% power. Allowing for attrition, recruiting to higher study numbers may be required.

### Statistical Analysis Plan

The analysis will be performed by the trial statistician with the statistical software R and WinBUGS. The trial statistician is blinded for the time up to database closure, and thereafter blinded to the specific treatment arms. For each of the primary, secondary and long-term outcomes, the null hypothesis is that there is no true difference in effect between the two trial treatments. The primary analysis will be performed using the complete case data only, i.e., excluding those participants with one or more missing records relevant for the particular analysis in question. For the primary and other analyses, those participants administratively or otherwise censored are not considered to have missing data in this context; their censoring date will be considered in the respective analysis.

#### Main Analyses

Primary analyses will be based on the intention to treat (ITT) principle. The ITT population includes all randomized participants, irrespective of treatment received, including rescue treatments. The flow of participants through the trial will be summarized using a CONSORT diagram. The number and percentage of participants with data until discharge/death/day 28 and during follow-up will be reported. Participant characteristics (e.g., age, gender, laboratory, and clinical characteristics before randomization) will be described for all participants, and separately for those randomized to each treatment arm. The number of days in hospital will be summarized with frequency tables, stratified by the randomized trial treatment.

The primary analysis will compare the log-transformed time to discharge/death/day 28 between the two trial treatments using a two-sided *t*-test, assuming unequal variance. Analogously to the pediatric arms of the U.K. RECOVERY trial, a supplementary Bayesian analysis of the length of stay will be performed comparing the two trial treatment arms with posterior mean estimates 95% credibility intervals reported. For time to event analyses, the log-rank test, respectively Gray's test, will be used to compare the differences between the arms. Cause-specific and Fine-Gray type subdistribution multivariable-adjusted Cox Proportional Hazards models will be fitted to investigate baseline covariate effects on time to event outcomes, assuming adequate numbers of events occur for these analyses to be sufficiently powered. The proportional hazards (PH) assumptions will be tested for these models, and, based on both visual checking and formal testing, time-dependent hazard approaches adopted in cases in which the PH assumption is deemed not to hold. The effect of time-varying independent variables (assuming missingness in these longitudinally captured variables is <30 %): CRP, D-Dimers, NT-pro-BNP, Troponin T, Fibrinogen, INR (coagulation), leucocytes, ejection fraction, and coronary artery aneurysm (diameter) will also be investigated. For the binary outcomes (e.g., need for inotropes, post-baseline use of ECMO), the risk ratio and absolute risk difference will be calculated with confidence intervals and *p*-value reported. Uni- and multivariable-adjusted logistic regression models will be fitted with dependent variables, the respective endpoint and independent variables (e.g., age, gender, ethnicity), and the time-varying variables (time to event analysis, for cardiac secondary outcomes only, as appropriate).

#### Sensitivity and Subgroup Analyses

Several pre-planned sensitivity and subgroup analyses will be performed. As defined above, the analyses will firstly be performed on the intention to treat population. In a further step, a supplementary analysis based on the “on treatment” population will be performed according to the actual treatment received, including those participants receiving a rescue treatment, if given after randomization. Any rescue treatment received, instead of, or in addition to, the randomized treatment until discharge/death/or day 28 will be reported.

Pre-planned subgroups analyses will be performed for different PIMS-TS phenotypes (PIMS-TS with shock, PIMS-TS with Kawasaki-Disease-like presentation, and for unspecified PIMS-TS) with respect to the primary and secondary outcome.

As described previously, analyses will generally be based on the complete case data. If deemed appropriate for specific analyses of post-discharge follow-up information including longer-term endpoints and/or time-dependent covariates, analyses of suitably multiply imputed data will be performed under the missing at random (MAR) assumption. The results will be presented in comparison with those from the complete case analysis, and any discrepancies discussed in the corresponding publications. If the MAR assumption seems to be inplausible then suitable sensitivity analyses will be considered. Allowance for multiple treatment comparisons will be made, as appropriate. For frequentist statistics, a *p*-value of <5% will be considered statistically significant throughout (unless stated otherwise). Any post-hoc analysis requested by oversight committees, a journal editor, or referees will be labeled explicitly as such. Any further future analyses not specified in the analysis protocol will be exploratory in nature and will be declared as post-hoc in any published reports.

In addition, we will provide graphs to describe temporal changes in physiological measures (fever, heart rate, systolic blood pressure), and laboratory markers (C-reactive protein, ferritin, troponin), comparing the steroid group vs. the IVIG group, starting at the time of randomization.

### Ethics

Before the trial initiation, the study protocol, the proposed patient information and consent forms, and other study-specific study documents were approved by the lead CEC (Ethics Committee Northwest/Central Switzerland; EKNZ), and the other responsible CECs in Switzerland for the participating sites (Project ID: 2021-00362).

### Current Trial Status

The first patient first visit (FPFV) in the Swissped-Recovery trial was the 23rd June 2021. Last patient last visit (LPLV) is scheduled for December 2022. SIVs have been performed at all 10 sites, with patients recruited from eight sites in Switzerland so far.

## Significance

This will be one of the first trials to compare the effectiveness of the two most widely used anti-inflammatory interventions to treat PIMS-TS. It will thereby address an urgent knowledge gap on whether there is an advantage to treat patients with PIMS-TS with IVIG at a dose of 2 g/kg/dose compared to 3 days of IV methylprednisolone 10 mg/kg/dose. The study design is informed by the U.K. RECOVERY trial. Future use of the Swissped RECOVERY trial data in joint analyses with the U.K. RECOVERY trial is planned. In addition to early clinical outcomes, such as the hospital length of stay, which are relevant for patients and healthcare services, the study includes several exploratory outcomes as proxies of the intervention effectiveness, as well as data on long-term cardiac and behavioral/functional assessment. In addition, follow-up until 6 months post-randomisation is collected. In summary, this study is anticipated to provide clinically directive evidence for optimal treatment of children with PIMS-TS.

## Ethics Statement

The studies involving human participants were reviewed and approved by the lead CEC (Ethics Committee Northwest/Central Switzerland; EKNZ), and the other responsible CECs in Switzerland for the participating sites (Project ID: 2021-00362). Written informed consent to participate in this study was provided by the participants' legal guardian/next of kin.

## Swissped Recovery Group

Kantonsspital Aarau, Aarau Switzerland: Spyridoula Gysi, MD; Indra Janz, MD; Andreas Bieri, MD.

University Children‘s Hospital Basel (UKBB), University of Basel, Basel, Switzerland: Birgit Donner, MD; Jürg Hammer, MD; Ulrich Heininger, MD; Clemens von Kalckreuth, MD; Malte Kohns, MD; Nicole Mettauer, MD; Alexandra Meyer; Diana Reppucci; Chloé Schlaeppi, MD; Daniel Trachsel, MD; Nina Vaezipour, MD; Andreas Woerner, MD; Andreas Zutter, MD.

Clinic of Pediatrics, Pediatric Institute of Southern Switzerland, EOC, Bellinzona, Switzerland: Lisa Kottanattu, MD; Calogero Mazzara, MD; Alessia Severi Conti, MD.

Inselspital, Bern University Hospital, University of Bern, Bern, Switzerland: Christoph Aebi, MD; Philipp Agyeman, MD; Andrea Duppenthaler, MD; Martin Glöckler, MD; Sabine Pallivathukal, MD; Thomas Riedel, MD.

University of Fribourg, Fribourg Hospital, Fribourg, Switzerland: Hong-Phuc Cudré-Cung, MD PhD; Mladen Pavlovic, MD.

Children's Hospital, Geneva University Hospitals and Faculty of Medicine, Geneva, Switzerland: Alice Bordessoule, MD; Anne-Laure Martin, MD; Angelo Polito, MD; Noemie Wagner, MD; Marie Rohr, MD; Arnaud L'Huilier.

University Hospital Lausanne, Lausanne, Switzerland: Vivianne Amiet, MD; Thomas Ferry, MD; David Longchamp, MD; Julia Natterer, MD; Rebecca Oppenheim, PhD; Michael Hofer, MD; Sabrina Bressieux-Degueldre, MD.

Children‘s Hospital, Hospital Lucerne, Lucerne, Switzerland: Katharina Wechselberger, MD; Alex Donas, MD; Sara Germann, MD; Michaela Lütolf Erni, MD; Daniela Kaiser, MD; Katharina Schwendener Scholl, MD; Hans Peter Kuen, MD; Katja Hrup, MD; Janine Stritt, MD.

Children's Hospital of Eastern Switzerland St. Gallen, St. Gallen, Switzerland: Tanja Wachinger, BSc; Ingrid Beck, MD; André Birkenmaier, MD; Bjarte Rogdo, MD; Philip Lorenz, MD; Ivo Iglowstein, MD; Konstanze Zöhrer, MD; Martin Flade, MD.

University Children's Hospital Zurich, University of Zurich (UZH), and Children‘s Research Center, Switzerland: Seraina Prader, MD; Jana Pachlopnik Schmid, PhD; Daniela Wütz, MD; Michelle Seiler, MD; Patrick Meyer Sauteur, PhD; Barbara Brotschi, MD; Kathrin Weber.

## Author Contributions

The study design originated from the U.K. RECOVERY Trial which was conceived by lead investigators Professors Martin Landray and Peter Horby, University of Oxford. SF is the Chair of the RECOVERY Trial Pediatric Working Group and with EW and colleagues in the UK initiated pediatric recruitment for RECOVERY and treatment arms focused on PIMS-TS. JB and LS designed this study, oversaw study setup, conduct, analyses setup, contributed to the first draft, approved the final version, and take responsibility for the accuracy of reported findings. TW and NS contributed to study design, setup, conduct, analyses setup, contributed to the first draft, and approved the final version. CS is the data manager for Swissped RECOVERY and performed interim analysis as mandated by the IDMC. AA wrote the statistical analysis plan. AA and CS contributed to study conduct and approved the final version. MA, DB, GB-R, MB, SG, HK, M-HP, JT, FV, and PZ are the local PIs or representants in the steering committee, performed patient recruitment, data collection, contributed to manuscript writing, and approved the final version. All authors contributed to the article and approved the submitted version.

## Funding

This work was supported by grants from the NOMIS Foundation, the Vontobel Foundation, and the Gaydoul Foundation (LS). Swiss PedNet (https://www.swisspednet.ch/) provides infrastructure support for study coordination, GCP, and monitoring.

## Conflict of Interest

AA is supported by the Swiss National Science Foundation grant CRSK-3_190977/1 and also works at Bern University Hospital. The remaining authors declare that the research was conducted in the absence of any commercial or financial relationships that could be construed as a potential conflict of interest.

## Publisher's Note

All claims expressed in this article are solely those of the authors and do not necessarily represent those of their affiliated organizations, or those of the publisher, the editors and the reviewers. Any product that may be evaluated in this article, or claim that may be made by its manufacturer, is not guaranteed or endorsed by the publisher.
